# Characterization of iPS87, a prostate cancer stem cell-like cell line

**DOI:** 10.18632/oncotarget.27524

**Published:** 2020-03-24

**Authors:** Erika N. Assoun, April N. Meyer, Maggie Y. Jiang, Stephen M. Baird, Martin Haas, Daniel J. Donoghue

**Affiliations:** ^1^Division of Biological Sciences, University of California San Diego, La Jolla, San Diego, CA 92093, USA; ^2^Department of Chemistry and Biochemistry, University of California San Diego, La Jolla, San Diego, CA 92093, USA; ^3^Department of Pathology, University of California San Diego, La Jolla, San Diego, CA 92093, USA; ^4^Moores UCSD Cancer Center, University of California San Diego, La Jolla, San Diego, CA 92093, USA

**Keywords:** prostate cancer, stem cells, androgen independent, androgen deprivation therapy, castration resistant prostate cancer

## Abstract

Prostate cancer affects hundreds of thousands of men and families throughout the world. Although chemotherapy, radiation, surgery, and androgen deprivation therapy are applied, these therapies do not cure metastatic prostate cancer. Patients treated by androgen deprivation often develop castration resistant prostate cancer which is incurable. Novel approaches of treatment are clearly necessary.

We have previously shown that prostate cancer originates as a stem cell disease. A prostate cancer patient sample, #87, obtained from prostatectomy surgery, was collected and frozen as single cell suspension. Cancer stem cell cultures were grown, single cell-cloned, and shown to be tumorigenic in SCID mice. However, outside its natural niche, the cultured prostate cancer stem cells lost their tumor-inducing capability and stem cell marker expression after approximately 8 transfers at a 1:3 split ratio. Tumor-inducing activity could be restored by inducing the cells to pluripotency using the method of Yamanaka. Cultures of human prostate-derived normal epithelial cells acquired from commercial sources were similarly induced to pluripotency and these did not acquire a tumor phenotype *in vivo*. To characterize the iPS87 cell line, cells were stained with antibodies to various markers of stem cells including: ALDH7A1, LGR5, Oct4, Nanog, Sox2, Androgen Receptor, and Retinoid X Receptor. These markers were found to be expressed by iPS87 cells, and the high tumorigenicity in SCID mice of iPS87 was confirmed by histopathology. This research thus characterizes the iPS87 cell line as a cancer-inducing, stem cell-like cell line, which can be used in the development of novel treatments for prostate cancer.

## INTRODUCTION

The American Cancer Society advises that prostate cancer is the second leading cause of cancer death for men in America. Prostate cancer is most common in men ages 65 and above. Despite approximately 175,000 new prostate cancer diagnoses occurring in the U. S. every year [1], prostate cancer screening via PSA measurements remains a controversial medical practice. In 2012, the U. S Preventive Services Task Force recommended the cessation of PSA testing for asymptomatic patients because the testing had resulted in the overtreatment of prostate cancer. However, current data has shown that the recommendations of the U. S. Preventative Task Force may have resulted in an increase of higher grade and more invasive malignant prostate cancer cases of Gleason score 8+. On recurrence, these cases are basically untreatable. Thus, new treatment paradigms for prostate cancer are urgently needed to reduce prostate cancer mortality.

Despite much research and clinical work, the identity of the prostate cancer cell, its initiation, and disease recurrence remain poorly understood. Recurrent prostate cancer mostly remains an incurable disease due to an incomplete and possibly incorrect understanding of its biological origins. We hypothesize that the disease is initiated, maintained, and progresses, as a disease of prostate cancer stem cells (PCSC) located in the outer/basal layer of the prostate glands [2, 3]. PCSC are significantly resistant to radiation, chemotherapeutic agents, and hormonal intervention. Clinical treatment of prostatic adenocarcinoma mostly recognizes and targets the more differentiated cells which, although derived from the immortal cancer stem cells, may no longer be immortal themselves. This approach may postpone disease progression, but recurrent/metastatic prostate cancer is nevertheless non-curable, due to failure to eliminate the underlying cancer stem cell population. To develop effective treatment approaches for prostate cancer, it is essential to understand the mechanisms responsible for the high rate of progression to incurable malignancy [2].

Previous publications from our laboratory have suggested that, at early points in prostate cancer diagnosis such as initial biopsy or prostatectomy, the cancerous prostate comprises the proliferation of PCSC [2, 3]. Patient needle-biopsy samples and prostatectomy tissue were examined for six stem cell-specific cell markers: CD44, CD133, Oct4, ALDH7A1, Nanog and LGR5, to verify the stem cell nature of the cancerous cells/tissues [2]. Antibody-stained prostate cancer tissue taken at the time of prostatectomy was indistinguishable from the images obtained from the same cancer tissue stained with H&E [2], suggesting that the prostate cancer cells at the time of prostatectomy are mostly composed of classes of stem cells. Thus, the appearance of H&E stained prostatic adenocarcinoma was identical to the appearance of parallel stem cell marker-stained tissue. From these experiments, we concluded that the earliest form of prostate cancer consists of the unscheduled proliferation of stem cells.

We have previously shown that prostate cancer originates as a stem cell disease [2, 3]. A prostate cancer patient sample, #87, obtained from prostatectomy surgery, was collected and frozen as a single cell suspension. Cancer stem cell cultures were grown, single cell-cloned, and shown to be tumorigenic in SCID mice [3]. However, outside its natural niche, the cultured PCSC lost their tumor-inducing capability and stem cell marker expression during tissue culture passage. Tumor-inducing activity was restored by inducing the cells to pluripotency using the method of Yamanaka [4]. Yamanaka factors (Oct3/4, Sox2, Klf4, c-Myc) are highly expressed in embryonic stem (ES) cells, and their over-expression has been shown to induce pluripotency and immortalization in both mouse and human somatic cells. These factors regulate the signaling network necessary for ES cell pluripotency and, typically, human iPSCs express stem cell markers and are capable of producing differentiated cells characteristic of all three germ layers [4–6]. In the work described here, we employed this approach towards a slightly different goal: to immortalize and restore tumorigenic properties to human PCSC. These restored iPS cells, designated iPS87, are characterized here. Our experiments suggest that prostate cancer is primarily propagated as a stem cell-associated disease and the iPS87 cell line provides a new opportunity to study this disease.

## RESULTS

### Tumorigenicity of the iPS87 cell line

**Figure 1:**
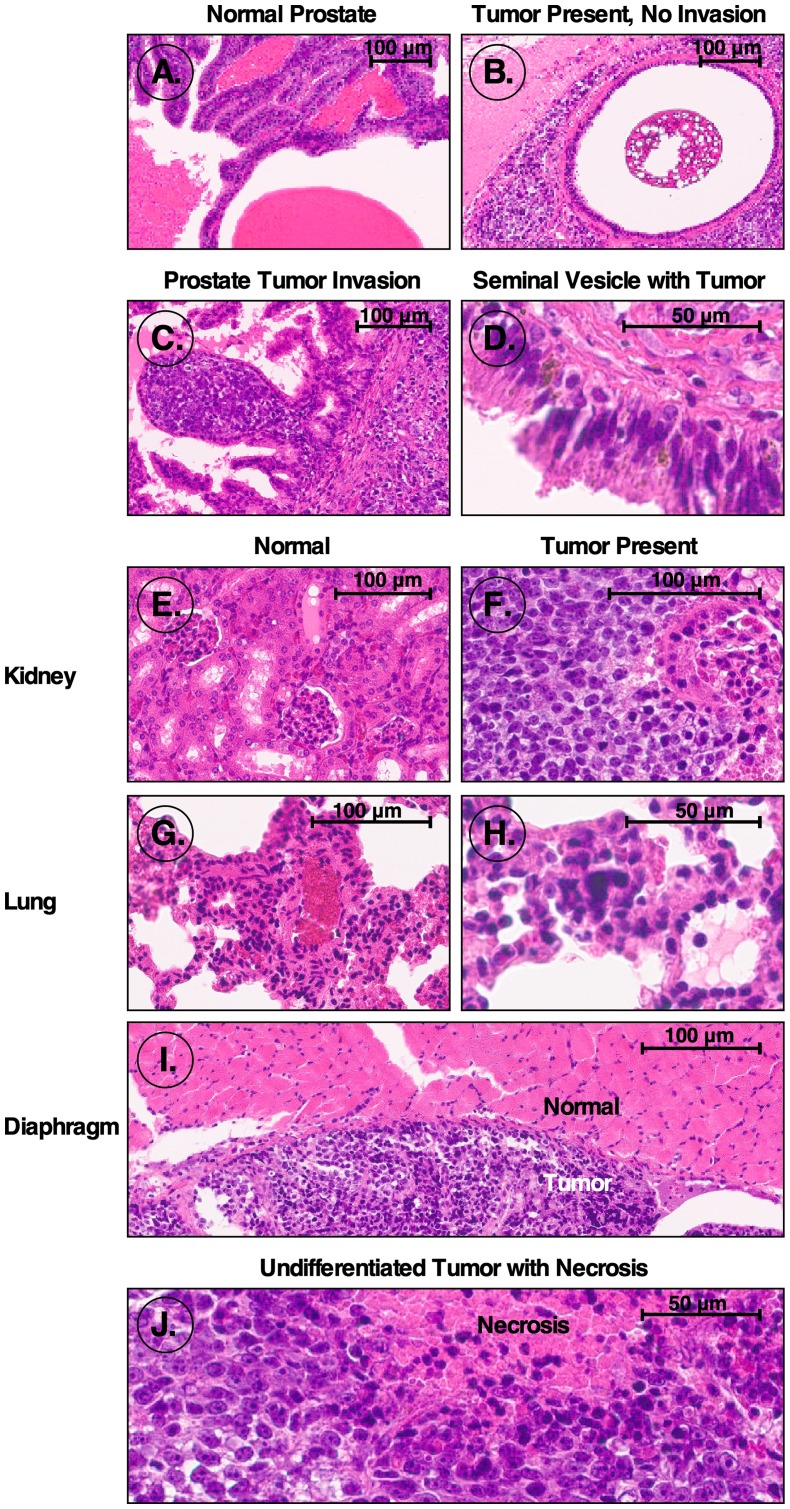
H&E stained sections from mice orthotopically injected with iPS87 cells. H&E stained sections were examined to identify tumor invasion. (**A**) Normal prostate; (**B**) non-invading tumor in prostate; (**C**) tumor invasion of prostate; (**D**) seminal vesicle tumor; (**E**) normal kidney; (**F**) tumor invasion of kidney; (**G**) normal lung; (**H**) lung tumor; (**I**) normal diaphragm (top) and tumor adjacent to diaphragm (bottom); (**J**) undifferentiated tumor mass with necrosis.

To determine the tumorigenicity of iPS87 cells, 10,000 iPS87 cells embedded in collagen were orthotopically implanted into the prostates of 24 SCID mice. Mice were observed and weighed weekly for approximately 90 days and sacrificed when tumor growth was determined based on total body mass, physical palpation, and visible abdominal tumor appearance. Mice with tumors usually exhibited significant secondary spreading typically involving the entire abdomen. Organs were fixed, embedded in paraffin and sectioned. Section slides were stained with H&E, and sections from three separate mice were taken and examined. These sections exhibited tumors in many abdominal organs. Figure 1A depicts normal mouse prostate tissue with apparent semen concretions. Figure 1B depicts a different section of the prostate tissue, from the same mouse as in Figure 1A, showing a non-invasive tumor. Furthermore, Figure 1C presents an area of tumor invasion within the prostate. A non-involved tumor was also identified within a seminal vesicle containing Lipofuscin granules, shown in Figure 1D.

Moreover, tumor tissue was identified within kidney, lung, and diaphragm tissues. Normal kidney tissue containing multiple glomeruli is depicted in Figure 1E, and an example of tumor invasion of the kidney and glomerulus is demonstrated in Figure 1F. The tumor invasion can be identified via the dark purple nuclei with distinctly prominent nucleoli characteristic of tumor cells. Additionally, Figure 1G shows normal mouse lung tissue with bronchioles and one blood vessel visible. Pulmonary emboli of mouse lung tissue with the start of a tumor center can be seen in Figure 1H. Figure 1I shows the diaphragm with the top-most region indicating normal tissue, and the bottom portion depicting a non-invasive tumor. Finally, Figure 1J depicts an anaplastic, undifferentiated, germ cell-like tumor, in which the cells reveal no cytological features associated with more differentiated tumors. Regions with apparent necrosis are also apparent. With this diagnostic evidence supporting the presence of tumors within the SCID mice transplanted orthotopically with iPS87 cells, it is evident that the iPS87 cell line is tumorigenic.

### Proliferation characteristics of iPS87 cells

**Figure 2:**
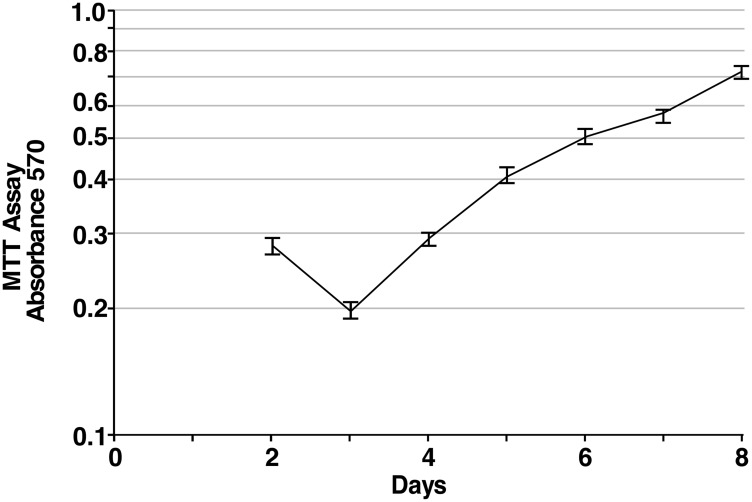
Proliferation of iPS87 cells. The average A570 of MTT treated iPS87 cells was measured daily in triplicate starting on Day 2 when cells had settled onto the feeder layer. The average value of daily triplicate values of MEF cells alone was subtracted to correct for MTT activity of the feeder layer. Standard deviation is shown.

In order to quantify the proliferation of iPS87 cells in culture, an MTT assay was performed with the results presented in Figure 2. iPS87 cells were plated at a low density and measured for proliferation for 8 days by MTT assay. From Days 3–5 when proliferation occurred at a maximal rate, a doubling time of approximately 42 hours was calculated. The iPS87 prostate cancer pluripotent stem cells have been cloned and continually transferred in culture for 4 years, corresponding to hundreds of cell doublings and thus can be considered to be immortal.

### Stem cell characteristics of iPS87 cells

Having presented *in vivo* data demonstrating the tumorigenicity of the iPS87 cell line (Figure 1), further characterization of the iPS87 prostate cancer cells was pursued using antibodies to known stem cell and receptor markers. Specifically, three common pluripotency markers, Oct 4, Sox 2, and Nanog, along with other stem cell markers, ALDH7A1 and LGR5, were selected [7–9]. ALDH7A1 is well known for its expression within the prostate, and LGR5 in the intestines [10, 11]. In addition to common stem cell markers, the Androgen Receptor (AR) and Retinoid X Receptor alpha (RXRα) were examined to determine their status.

**Figure 3:**
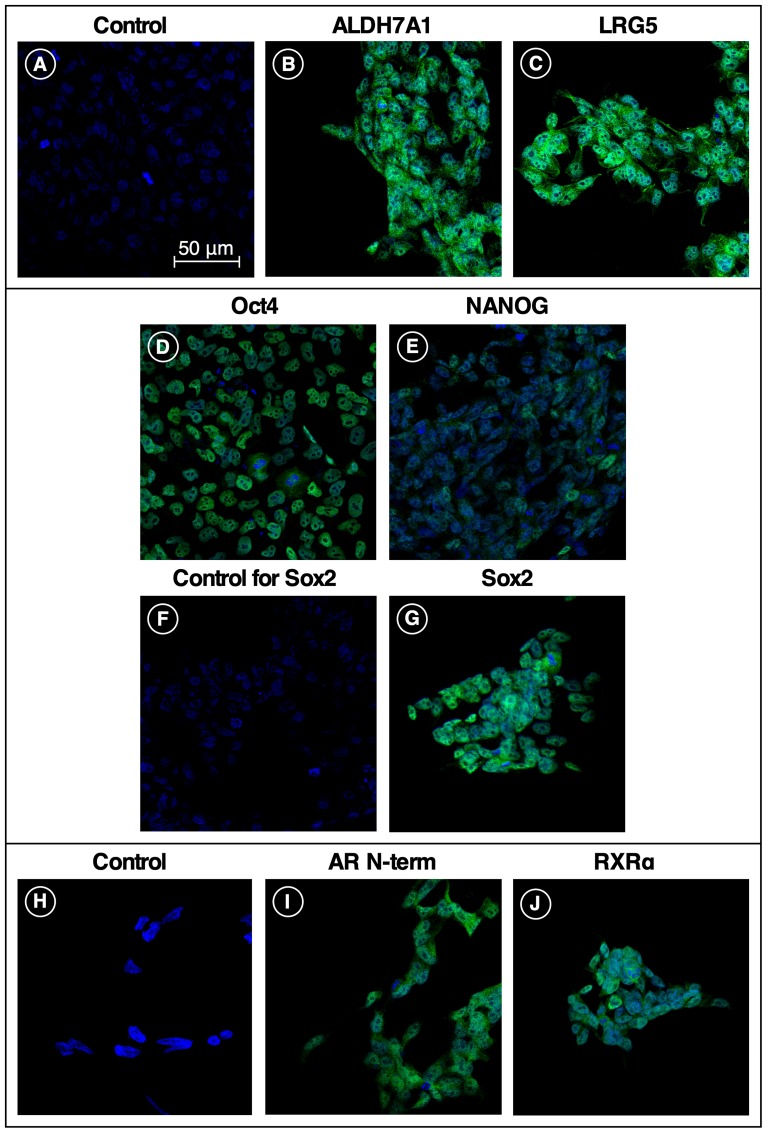
Stem cell and receptor markers expressed in iPS87 cells. Five stem cell markers and two receptor markers were tested for presence in iPS87 cells by indirect immunofluorescence: (**A**) secondary-only control; (**B**) ALDH7A1; (**C**) LGR5; (**D**) Oct 4; (**E**) Nanog; (**F**) secondary-only control; (**G**) Sox 2; (**H**) secondary-only control; (**I**) Androgen Receptor N-Terminus; and (**J**) RXRα. All images at same magnification, with 50 μM scale bar shown in (A).

Figure 3 illustrates the immunofluorescent staining of fixed iPS87 cells with antibodies to stem cell markers and prostate cell markers ALDH7A1, LGR5, Oct4, Sox2, and Nanog. ALDH7A1 (Figure 3B) and Sox2 (Figure 3G) had prominent cytoplasmic and nuclear staining, while LGR5 (Figure 3C), Oct4 (Figure 3D), and Nanog (Figure 3E) staining were observed to be mostly cytoplasmic. Furthermore, immunofluorescent staining of Androgen Receptor (Figure 3I) similarly showed prominent cytoplasmic staining. Lastly, RXRα (Figure 3J) was observed as both cytoplasmic and nuclear staining.

In summary, with the positive staining of five documented stem cell markers, we conclude that the iPS87 cell line is indeed stem cell-like. The expression of the Androgen Receptor suggests that the iPS87 cells possess a stem cell progenitor- or a stem cell transit-amplifying genotype. This could potentially facilitate studies of the responsiveness of this potently tumorigenic cell to Androgen Deprivation Therapy (ADT).

## DISCUSSION

### Prostate cancer metastasis

Prostate cancer is known to metastasize to bone and lymph nodes, but the mechanisms of metastasis are unknown [12]. We found that iPS87 prostate tumor derived stem cells are highly tumorigenic. Figure 1 illustrates the regions in which tumors developed after orthotopic transplantations of iPS87 cells into the prostate. Interestingly, the tumors did not invade the lungs; however, this could be due to the lung lacking the proper niche or microenvironment for these cells to replicate (Figure 1H). A similar non-invasive pattern is found with ovarian carcinomas. Ovarian cancer often metastasizes to the peritoneal cavity, and simply attaches to organs including the gut without invasion, but the mechanisms are not fully understood [13]. We would suggest that, upon dissemination to distant locations, prostate carcinoma cells can grow in lymph nodes and bone as these organs have a suitable niche that supports the proliferation of prostate cancer stem/progenitor cells. This or similar mechanisms may be responsible for the typical dissemination of specific cancers, because – in prostate and potentially many other cancers – the cells that metastasize to distant locations are stem/progenitor cells which require a specific niche for self-renewal and/or differentiation. In the current case, mouse iPS87 stem cell tumors did show some sites of invasion within the prostate and kidney (Figure 1C, 1F).

### Presence and localization of 5 stem cell markers in iPS87 cells

We further found that five stem cell markers are expressed by the highly tumorigenic iPS87 stem cell line: ALDH7A1, LGR5, Oct 4, Nanog, and Sox 2. ALDH7A1, an isoform of aldehyde dehydrogenase, and a known breast cancer stem cell marker, assists in the breakdown of retinal to retinoic acid, aiding in the differentiation of breast stem cells. ALDH7A1 is also a known cancer stem cell marker for multiple myeloma, acute leukemia, and brain tumors [14]. It has been shown that when ALDH7A1 is knocked down, the stem cell progenitor subpopulation in a particular prostate cancer cell line decreased [10]. ALDH7A1 has also been shown to be involved in the process of prostate cancer bone metastasis [10]. The presence of ALDH7A1 in Figure 3B appears as both cytoplasmic and nuclear localized. This corresponds to common observations of ALDH7A1 in subcellular compartments including the cytosol, nucleus, and mitochondria [15].

LGR5 is a G Protein-Coupled Receptor that has been classified as a colorectal, liver, and intestinal stem cell marker [11, 16, 17]. Lineage tracing studies in liver have shown that LGR5 stem cells aid in the formation of tumor [17]. Furthermore, other studies have determined that the presence of LGR5 in cervical cancer is indicative of enhanced self-renewal capacity, differentiation, and tumorigenicity [16]. We have previously shown that LGR5 is present in prostate cancer [2]. Moreover, Figure 3C depicts prominent cytoplasmic staining of LGR5 in iPS87 stem cells. This localization correlates with the cytoplasmic localization of LGR5 within a colon cancer stem cell [18].

Oct 4, a transcription factor and common pluripotent stem cell marker, which is composed of both Oct 4A and Oct 4B, is thought to have different functions. Oct 4A, commonly found in the nucleus of a cell, is responsible for the pluripotency of embryonic stem cells, while Oct 4B resides in the cytoplasm in cervical cells and is thought to regulate the progression of cervical cancer among other cancer related functions [7, 19]. Figure 3D reveals primarily cytoplasmic staining, suggesting that Oct4B may be the dominant Oct4 form found within the iPS87 cell line.

Sox 2 is a well-known transcription factor that among other roles is responsible for maintaining the pluripotency and self-renewal of embryonic stem cells [8, 20]. It is known to interact with various proteins, including Oct 4, and the Sox2-Oct 4 interaction is believed to maintain pluripotency and repress developmental processes [8, 21]. Immunocytochemical analysis of esophageal squamous cell carcinoma and prostate cancer showed Sox 2 localization within the nucleus [22–24]. Although the mechanism of nuclear export of Sox 2 is not well understood [25], we observed both nuclear and cytoplasmic staining in our study (Figure 3G).

Nanog, a homeobox transcription factor, is a pluripotent stem cell marker that is present in embryonic stem cells and has been thought to maintain self-renewal and undifferentiation of PCSC [9, 26]. Nanog has been found in colorectal, breast, cervix, ovarian, lung, and head and neck tumors as well [27, 28]. Immunofluorescent and immunocytochemical analysis of prostate cancers previously identified strong nuclear and some cytoplasmic staining of Nanog [9, 27]. Presence of cytoplasmic Nanog was found in mesenchymal stem cells in cervical cancer [29], and researchers have found that Nanog localization is strongly associated with cell type. Furthermore, researchers identified that stromal cytoplasmic staining of Nanog is related to the advancement of cervical cancer. The Nanog staining we see in iPS87 cells (Figure 3E) was predominantly cytoplasmic. The localization of Nanog may be prostate cancer cell type-dependent; prostate cancer cell lines DU145 and PC3 cells show strong nuclear and some cytoplasmic Nanog staining; however, these cell lines are derived from prostate cancer metastases to brain and bone, offering the possibility that they represent a more differentiated cell type or malignant phenotype than iPS87 cells [29–31].

### Androgen receptor and androgen deprivation therapy

Androgen Receptor (AR) is a cytoplasmic steroid receptor composed of four domains, the N-terminal, transactivation, DNA binding, and ligand binding domains [32]. Androgen Receptor is located in the cytoplasm of a normal prostate cell when no androgens are present. Upon androgen binding, AR dimerizes, translocates into the nucleus, and initiates the transcription of various genes [32, 33]. However, in androgen refractory prostate cancer cells, regardless of the presence of androgen, the AR is nuclear localized [32]. Although the nuclear import and export of AR is not well established, it is known that the AR must be in the nucleus in order to induce transcription [32]. Figure 3I depicts dominant cytoplasmic staining of AR in iPS87 cells. The iPS87 cell line was originally developed in medium devoid of an androgen source, suggesting that the iPS87 cell line is androgen independent, and has not undergone the morphological changes of Castration Resistant Prostate Cancer (CRPC). Moreover, the presence of AR in iPS87 cells provides context to and support for the need to develop curative prostate cancer treatments.

Controversy over the use of ADT to treat metastatic prostate cancer is ongoing [34]. Since patients’ prostate cancer cells are thought to overexpress the AR, one basic treatment for recurrent systemic disease, is breaking the Androgen-AR pathway. Clinically, the pathway is broken by suppressing the availability of testosterone via the use of Luteinizing Hormone-Releasing Hormone (LHRH) agonists [35, 36]. This method inhibits Luteinizing Hormone (LH) release from the anterior pituitary, and results in the inability of Leydig cells to produce testosterone [37]. Effective suppression of recurrent prostate cancer cell proliferation by interrupting this pathway has necessitated the synthesis of multiple, specific and effective drugs with anti-hormone and/or anti-AR activity including estrogens, LHRH agonists, and more [36]. ADT has been successful, in that the treatments showed destruction via apoptosis or cell cycle death of prostate cancer cells [37].

However, even with ADT, the majority of patients with recurrent prostate cancer eventually succumb to their disease as alterations of the AR pathway can result in androgen independent mechanisms that lead to the progression of prostate cancer [37]. Although the medical profession has succeeded in designing combination treatments that prolong the lives of the patients by months or a few years, treatments are not curative. Thus, more research on the characterization and derivation of potently tumorigenic human PCSC is needed to understand the complete mechanism of prostate cancer initiation and recurrence.

### Retinoic acid receptor and possible differentiation treatment

Retinoid X Receptor/Retinoic Acid Receptors are a group of nuclear receptors that form heterodimers upon All Trans Retinoic Acid (ATRA) or ligand binding. When ATRA binds, the pathway elicits a response that leads to the transcription of various genes through the activation of RAF/MAPK pathways [38, 39]. We examined iPS87 cells for the presence of RXRα, one of the heterodimers of the complete complex. Today, ATRA is clinically used for the treatment of psoriasis and Acute Myelocytic Leukemia (AML). Studies have shown that even at low concentrations of ATRA, binding to the Retinoic Acid or Retinoid Receptors leads to the differentiation or cell cycle arrest of the HL-60 cell line within 48 hours [38, 40]. With the presence of RXRα in the iPS87 cell line, we were interested to examine the effects of ATRA treatment. We surmised that even small doses and short time frames might have profound effects by promoting differentiation. Nevertheless, iPS87 cells were resistant to concentrations of ATRA as high as 10 μM. However, the differentiative nature, and availability of many synthetic retinoids should be further studied, as the iPS87 cell line or other patient-derived PCSC lines may undergo differentiation in response to these ligands.

## MATERIALS AND METHODS

### Cell culture

A Mouse Embryonic Fibroblast (MEF) feeder layer was prepared as described [41]. MEFs were grown on 0.1% gelatin in coated tissue plates, and treated with 10 μg/ml Mitomycin C (Alfa Aesar Catalog: J63193) for 3 hours after reaching 30% confluence. Cells were rinsed with PBS and maintained in DMEM (Gibco) supplemented with 10% Fetal Bovine Serum (Hyclone, Thermo Scientific), 2% L-glutamine (Life Technologies), 1% Sodium Pyruvate (Corning) 1% Fungizone/amphotericin (Biologos)/0.5% gentamycin (Elkins-Sinn, Inc.) or 1% antimycotic (Gemini), and 1% non-essential amino acids (Gibco), referred to as “D10”. The cells were incubated at 6% CO_2_ and 37°C until iPS87 cells were ready to be transferred (1–2 days). For the MTT assay, MitC-treated MEFs from Thermo Fisher Scientific were used per manufacturer’s protocol (Gibco, A34959).

Human prostate cancer epithelial cells grown from prostatectomy-derived cells of patient #87 [3] and induced to perpetual stem-cell pluripotency using the retroviral vector plasmids pMXs-hOCT4, pMXs-hSOX2, pMXs-hKlf4, pMXs-hc-Myc. Cells were transduced by spinfection [42]. Induced Pluripotent Stem cells (iPS) from the cultured epithelial cells of patient #87 were single cell-cloned three times in succession, then frozen for future use. For later analysis, the iPS-87 cells were grown on Mitomycin-C inactivated MEF feeder cells and maintained in KnockOut DMEM (Gibco) supplemented with 0.125% Bovine Serum Albumin (Sigma), 2% L-glutamine, 1% non-essential amino acids, 1% Fungizone/0.5% gentamycin or 1% antimycotic, 10% serum replacement (Gibco), 6.25 ng/mL bFGF (Peprotech), referred to as “ES++,” 6% CO_2_, 37°C.

### Sectioning and immunocytochemistry

10^4^ iPS87 cells in collagen were implanted into the prostate of SCID mice under Animal Care Protocol S07410. Orthotopic implantation was performed as previously described [3]. Mice were weighed weekly over a period of 10-12 weeks and examined physically and by palpation. Unambiguous abdominal tumors typically manifested between 7–9 weeks. Once sacrificed, organs were fixed for 24 hours and provided to the Tissue Technology Shared Resource at UCSD’s Moore’s Cancer Center to be embedded in paraffin, sectioned, and stained with H&E. Slides were scanned by the Tissue Technology Shared Resource Center and imaged on Aperio Image Scope software at magnification varying from 10× to 40 ×.

### MTT assay

Plate were set up with a layer of mouse embryonic fibroblast feeder cells at 3 × 10^5^ cells per 35 mm plate. The next day, a 60 mm plate of ~90% confluent iPS87 cells was seeded 1:1000 per 35 mm plate. 500 μg/mL of 3(4,5-dimethylthiazol-2-yl)-2,5-diphenyltetrazolium bromide (MTT, Sigma), was added to plates containing iPS87 cells, and incubated for approximately 2 hours in 5% CO_2_ at 37°C. Control samples of ES++ media only and MEF only were used. Dye was solubilized with an equal volume of 40 mM HCL/isopropanol for 30 min at 5% CO_2_, 37°C. Readings were taken daily, and triplicate plates were read in triplicate at absorbances of 570 nm and 630 nm.

### Immunofluorescence

For immunofluorescence experiments, when the MEF layer grown in 24-well plates containing glass cover slips coated with 0.1% gelatin reached 30% confluence, iPS87 cells were plated at a density of 2.0 × 10^4^ cells per well. These cells were maintained at 6% CO_2_, 37°C, rinsed with PBS, and fixed with 4% para-formaldehyde/PBS. All fixed cells were rinsed with PBS. Coverslips were stored in 50% glycerol/PBS at −20°C. Coverslips were washed with PBS, permeabilized with 0.1% Triton-X-100 in PBS at RT for 15 min, washed with PBS, and blocked with 5% goat or donkey serum/PBS at RT for 45 minutes. The control samples were incubated with PBS during the primary antibody incubation step. All other samples were stained with one of the following antibodies at a 1:100 dilution in either 5% goat or donkey serum for 2 hours at room temperature in a dark humidified chamber. Antisera used were as follow: ALDH7A1 AntiPicoband (ABO11656) from Abcam; Oct 4 (GTX101497), LGR5 (GTX129862), Nanog [N3c3] (GTX100863), and Androgen Receptor [N1] N-terminus (GTX10056) from Genetex; Sox2 [E4] (SC-365823) and RXRalpha (SC-553) from Santa Cruz Biotech. Coverslips were then rinsed thoroughly with PBS and the appropriate secondary antibody, diluted at 1:1000 in either 5% goat or donkey serum, placed on the coverslip and incubated in a dark humidified chamber for one hour. The following secondary antibodies were used: Alexa Fluor 488 Goat anti-rabbit (Jackson Immuno Research 111-545-003) and Alexa Fluor 488 Donkey anti-mouse H+L IgG (Invitrogen A21202). Coverslips were rinsed thoroughly with PBS, and 300 nM DAPI (Molecular Probes) diluted in PBS was placed on each coverslip for 10 minutes in a dark humidified chamber. Coverslips were rinsed thoroughly with PBS, and mounted with ProLong Gold Antifade Reagent with DAPI (Invitrogen P36935) on microscope slides and stored at 4°C.

### Microscopy

Stained coverslips were imaged at the UC San Diego Microscopy Core (Grant NS04710) using a Zeiss 880 Airyscan Confocal Microscope. Zen Black Airyscan Image Processing Software version 2.3 SP1 was used to process the images.

## Abbreviations

ADT: Androgen Deprivation Therapy
ALDH7A1: Aldehyde Dehydrogenase 7 Family Member A1
AML: Acute myelocytic leukemia
AR: Androgen Receptor
ATRA: All-trans retinoic acid
CRPC: Castration Resistant Prostate Cancer
DU145: prostate cancer cell line
H&E: hematoxylin and eosin
iPS: induced pluripotent stem cells
LGR5: Leucine Rich Repeat Containing G Protein Coupled Receptor 5
LH: Luteinizing Hormone
LHRH: Luteinizing Hormone-Releasing Hormone
LNCaP: prostate cancer cell line
MAPK: Mitogen Activated Protein Kinase
MTT: 3-(4.5-Dimethylthiazol-2-yl)-2-5-diphenyltetrazolium bromide
Nanog: Homeobox Transcription Factor Nanog
Oct4: Octamer-Binding Protein 4; also POU Domain, Class 5, Transcription Factor 1
PC3: prostate cancer cell line
PCSC: Prostate Cancer Stem Cell
PSA: prostate specific antigen; also, Kallikrein Related Peptidase 3
RXRα: Retinoid X Receptor alpha
SCID: Severe combined immunodeficiency
Sox2: Sex determining region Y box 2
